# A genome-wide association study of serum uric acid in African Americans

**DOI:** 10.1186/1755-8794-4-17

**Published:** 2011-02-04

**Authors:** Bashira A Charles, Daniel Shriner, Ayo Doumatey, Guanjie Chen, Jie Zhou, Hanxia Huang, Alan Herbert, Norman P Gerry, Michael F Christman, Adebowale Adeyemo, Charles N Rotimi

**Affiliations:** 1 Center for Research on Genomics and Global Health, National Human Genome Research Institute, Bethesda, MD 20892 USA; 2 Department of Genetics and Genomics, Boston University, Boston, MA 02118 USA; 3 Coriell Institute for Medical Research, Camden, NJ 08103 USA

## Abstract

**Background:**

Uric acid is the primary byprodu**c**t of purine metabolism. Hyperuricemia is associated with body mass index (BMI), sex, and multiple complex diseases including gout, hypertension (HTN), renal disease, and type 2 diabetes (T2D). Multiple genome-wide association studies (GWAS) in individuals of European ancestry (EA) have reported associations between serum uric acid levels (SUAL) and specific genomic loci. The purposes of this study were: 1) to replicate major signals reported in EA populations; and 2) to use the weak LD pattern in African ancestry population to better localize (fine-map) reported loci and 3) to explore the identification of novel findings cognizant of the moderate sample size.

**Methods:**

African American (AA) participants (*n *= 1,017) from the Howard University Family Study were included in this study. Genotyping was performed using the Affymetrix^® ^Genome-wide Human SNP Array 6.0. Imputation was performed using MACH and the HapMap reference panels for CEU and YRI. A total of 2,400,542 single nucleotide polymorphisms (SNPs) were assessed for association with serum uric acid under the additive genetic model with adjustment for age, sex, BMI, glomerular filtration rate, HTN, T2D, and the top two principal components identified in the assessment of admixture and population stratification.

**Results:**

Four variants in the gene *SLC2A9 *achieved genome-wide significance for association with SUAL (*p*-values ranging from 8.88 × 10^-9 ^to 1.38 × 10^-9^). Fine-mapping of the SLC2A9 signals identified a 263 kb interval of linkage disequilibrium in the HapMap CEU sample. This interval was reduced to 37 kb in our AA and the HapMap YRI samples.

**Conclusions:**

The most strongly associated locus for SUAL in EA populations was also the most strongly associated locus in this AA sample. This finding provides evidence for the role of *SLC2A9 *in uric acid metabolism across human populations. Additionally, our findings demonstrate the utility of following-up EA populations GWAS signals in African-ancestry populations with weaker linkage disequilibrium.

## Background

In humans, uric acid is the primary byproduct of purine metabolism and has long been associated with the development of gouty arthritis [1,2]. Since the late 1800 s, it has been postulated that hyperuricemia plays a role in gout, kidney dysfunction, and vascular tone [3]. Over the past several decades, evidence linking uric acid to body mass index (BMI), insulin resistance, the metabolic syndrome, [4,5], dietary intake of food substances high in purine [1], dietary fructose intake [2,6,7], renal disease and hypertension [8-12] has been expanding.

Clustering of uric acid, gout, renal disease, and hypertension has been known to have familial links since the late 1800 s [11,12], suggesting a hereditary component to these traits. Furthermore, varying levels of uric acid in human populations, in addition to being attributable to dietary habits, are likely the result of evolutionary mutations that took place greater than 8 million years ago [13,14]. These mutations have lead to the genetic variation we see in modern human populations [1,15].

Mounting evidence generated from genome-wide association studies (GWAS) have linked uric acid to specific genomic loci [4,16-18]. However, the GWAS reporting association of uric acid with specific genetic loci (*PDZK1*, *GCKR*, *SLC16A9*, *SLC22A11*, *SLC22A12*, *LRRC16A*, *WDR1*, *RAF1P1*, *ZNF5188*, and *ABCG2*), have been conducted in individuals of European [4,17,18], and Asian [19] descent. Given the paucity of GWAS in populations of African-ancestry, we chose to focus this manuscript on three main objectives in the following order of priority 1) to replicate major signals for uric acid reported in EA populations; 2) to use the weak LD patterns in African-ancestry populations to better localize (fine-map) reported loci and 3) to explore the identification of novel findings cognizant of the moderate sample size as well as the higher rates of obesity, renal disease, T2D, HTN, and decreased glomerular filtration rate in African Americans [20].

## Methods

### Ethics Statement

Declaration of assurance of ethical conduct of research was granted by the Howard University Institutional Review Board. All participants provided written informed consent for specimen collection and analysis. This study adhered to the tenets of the Declaration of Helsinki.

### Study Sample

The study population has been described previously [21]. Briefly, participants included in this study were derived from the Howard University Family Study (HUFS), a population-based study of related and unrelated African Americans from the Washington, D.C. metropolitan area. The primary aims of HUFS included: 1) enrollment and examination of a randomly ascertained sample of 350 African American families with members in multiple generations from the Washington, D.C. metropolitan area; 2) characterization of participants for anthropomorphic measures (including height, weight, body composition measures and measures of obesity, blood pressure and related physiological intermediates, and diabetes-related and lipid-related variables); and 3) storing high-quality DNA to conduct studies to identify genes or genomic regions linked and/or associated with common, complex traits. Recruitment was conducted via door-to-door canvassing, community events, and advertisement in regional papers. A population-based approach was used to establish an unascertained sample with which to study multiple, common diseases. In a second phase of recruitment, additional unrelated individuals from the same geographic area were enrolled to facilitate nested case-control studies. Enrollment procedures (forms, measurements, and laboratory assays) for unrelated individuals were identical to those for families. The total number of recruited individuals was 2,028, of which 1,976 remained after data cleaning. From this sample, we created a subset of 1,055 unrelated adults (≥ 20 years of age).

### Phenotyping

A baseline physical examination and an interview-based demographic questionnaire were conducted. Blood was drawn for biochemical assays for creatinine, glucose, uric acid, and several other molecular phenotypes. Weight was measured on an electronic scale to the nearest 0.1 kg with the participant wearing light clothes. Height was measured with a stadiometer to the nearest 0.1 cm with participants in bare feet. Body mass index was calculated as (weight in kg)/(height in m)^2^. Blood pressure was measured while participants were seated using an oscillometric device (Omron Healthcare, Inc., Bannockburn, Illinois). The readings were taken with a ten minute interval between readings. Reported systolic and diastolic blood pressure readings were the averages of the second and third readings. Participants with systolic blood pressure ≥ 140 mm Hg, diastolic blood pressure ≥ 90 mm Hg, or on prescribed antihypertensive drug therapy were defined as hypertensive. Serum creatinine levels were estimated on fasting sample using the modified Jaffé method. Estimated glomerular filtration (eGFR) was calculated using the simplified Modification of Diet in Renal Disease Study equation: eGFR = 186 × (serum creatinine)^-1.154 ^× age^-0.203^(× 0.742 if female)(× 1.210 if Black) [22]. EGFR was measured in ml/min/1.73 m^2 ^and creatinine was measured in mg/dl. Participants with fasting plasma glucose ≥ 7.0 mmol (126 mg/dl) were defined as having type 2 diabetes. Individuals with prediabetes (fasting plasma glucose between 5.6 mmol and 7.0 mmol) were given unknown case/control status. Serum uric acid levels were determined using the COBAS Integra Uric Acid assay, version 2 (Roche Diagnostics, Indianapolis, Indiana).

### Genotyping

The Affymetrix^® ^Genome-Wide Human SNP array 6.0 was used to conduct genome-wide genotyping [23]. Genetic material was processed and hybridized according to the manufacturer's instructions. Following processing, the chips were scanned and genotype calls were determined using the Birdseed 2 algorithm [23,24]. The individual sample call rate had to be ≥ 95% for inclusion (no samples excluded). SNPs with call rates < 95% (*n *= 41,885) across all individuals, minor allele frequency ≤ 0.01 (*n *= 19,154), or a Hardy-Weinberg equilibrium (HWE) test *p*-value < 1 × 10^-3 ^(*n *= 6,317) were excluded. This analysis included the 808,465 autosomal SNPs that passed these filters. The average call rate for SNPs in this group of individuals was 99.5% and the agreement of blind duplicates was 99.74%.

### Imputation

Imputation of missing SNPs was performed using MACH, version 1.0.16 http://www.sph.umich.edu/csg/abecasis/MACH/[25] using a two stage approach. We downloaded the HapMap combined phase II+III raw genotype files for NCBI build 36, release 27 from http://hapmap.ncbi.nlm.nih.gov/downloads/genotypes/2009-02_phaseII+III/forward/non-redundant/[26]. For both the CEU and YRI samples, we retained only those individuals marked as founders. SNP inclusion criteria for imputation were that they had a MAF ≥ 0.01, a missingness rate ≤ 5%, and an individual missingness rate of ≤ 5%. These criteria resulted in 2,327,370 CEU and 2,598,198 YRI reference SNPs. Imputation was performed separately for these two reference panels. In the first stage, haplotype phases for the reference data were inferred using the settings -rounds 50 -states 200. In the second stage, imputation was conditioned upon the maximum-likelihood estimates of the crossover map and the error rate map. Imputation error was calibrated by ascertaining the threshold of posterior probability associated with a 10% error rate for the CEU reference panel and a 5% error rate for the YRI reference panel averaged over 6,800 SNPs for which we masked the experimentally determined genotypes. Imputed genotypes had to pass quality control filters requiring MAF ≥ 0.01, SNP missingness rate ≤ 10%, and HWE test *p*-value ≥ 0.001. For imputed genotypes that differed when using the CEU and YRI reference panels, we accepted the imputed genotype using the YRI reference panel. We successfully imputed 1,558,391 SNPs, yielding 2,366,856 experimentally determined and imputed autosomal SNPs. Quality control and data management were performed using PLINK, which is freely available and can be downloaded from http://pngu.mgh.harvard.edu/purcell/plink/[27]. More detailed descriptions of these procedures have been described previously [28,29].

### Assessment of Population Stratification

Assessment of population stratification was done via nonparametric clustering of genotypes using AWClust [30]. From the sample of 1,055 unrelated individuals, 37 individuals were identified as outliers and were excluded from analysis. Possible inflation of the type I error rate due to population stratification was investigated using genomic control [31]. EIGENSOFT was used to assess population structure [32]. A previously published scree plot [21] illustrates the two principal components (PCs) used as covariates in the analysis.

### Association Analysis

Of the 1,018 included participants, one was missing the serum uric acid measurement, leaving a final analyzed sample of 1,017 individuals. Descriptive and multivariate analyses were conducted using R version 2.10.0 [33]. Serum uric acid values were not normally distributed. Therefore, serum uric acid values were transformed using a Box-Cox transformation (Box-Cox parameter lambda = 0.54). In the multivariate analysis, serum uric acid was analyzed for association with potential covariates age, sex, BMI, T2D, HTN, and eGFR. In genetic analysis, serum uric acid was analyzed as a continuous variable using linear regression, adjusting for age, sex, BMI, HTN, T2D, eGFR, and the first two PCs of the genotypes as covariates. PLINK, version 1.07 [27], was used to conduct the association analyses.

## Results

**Figure 1:**
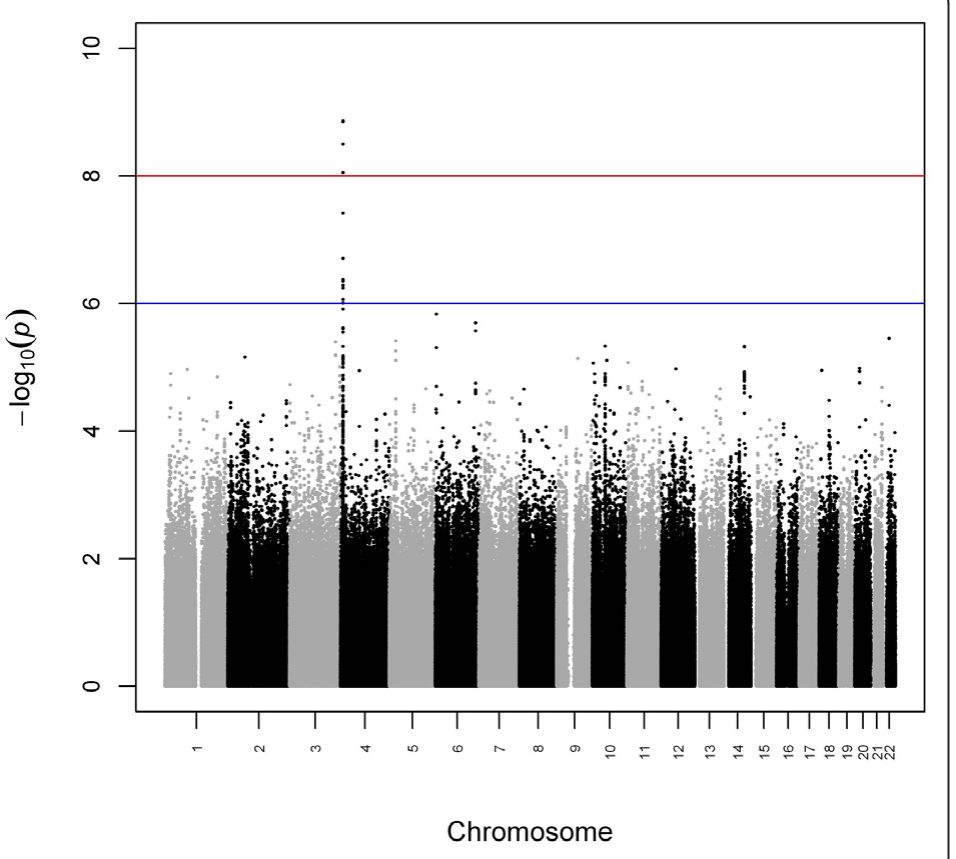
**Distribution of *p*-values from the multiple linear regression of SNPs, associated with uric acid**. The red line indicates the genome-wide significance level 1 × 10^-8 ^and the blue line indicates the suggestive significance level 1 × 10^-6^.

Clinical characteristics of the participants of our study are displayed in Table 1. In the univariate analysis, age (*p *= 4.68 × 10^-9^), sex (*p *= 4.83 × 10^-35^), BMI (*p *= 6.98 × 10^-7^), eGFR (*p *= 1.33 × 10^-26^), HTN (*p *= 6.93 × 10^-16^), and T2D (*p *= 0.021) were identified as significant covariates. In the multivariate linear regression analysis, sex, BMI, HTN, and eGFR (but not age and T2D) were significantly associated with uric acid levels (Table 2). The distribution of *p*-values for genetic association with serum uric acid is illustrated in Figure 1. The genomic control inflation factor was 0.9964, indicating no inflation of the type I error rate due to population stratification (Additional File 1). The top 25 ranked SNPs unadjusted for covariates are provided in Additional File 2 the 25 top ranked SNPs adjusted for age and sex are provided in Additional File 3 while the top 25 ranked SNPs adjusting for all covariates are provided in Additional File 4.

**Table 1:** Clinical characteristics of the African Americans included in this study

Variable	Mean (SD) or Percent
Age (yr)	48 (13)
Male	41%
Body Mass Index (kg/m^2^)	30.5 (8.3)
Hypertension	50%
Type 2 Diabetes	15.6%
Glomerular Filtration Rate (ml/min/1.73 m^2^)	105.1 (32.0)
Serum uric acid (mg/dl)	5.53 (1.64)

**Table 2:** Multivariate linear regression between covariates and serum uric acid levels

Variable	β	SE	*P*-value
Age	0.0008	0.0018	0.665
Sex	-0.6576	0.0405	8.30 × 10^-53^
Body Mass Index	0.0190	0.0025	2.82 × 10^-14^
Hypertension	0.2385	0.0441	8.17 × 10^-8^
Type 2 diabetes	-0.0161	0.0565	0.775
eGFR	-0.0072	0.0007	4.76 × 10^-26^

eGFR: estimated glomerular filtration rate

**Figure 2:**
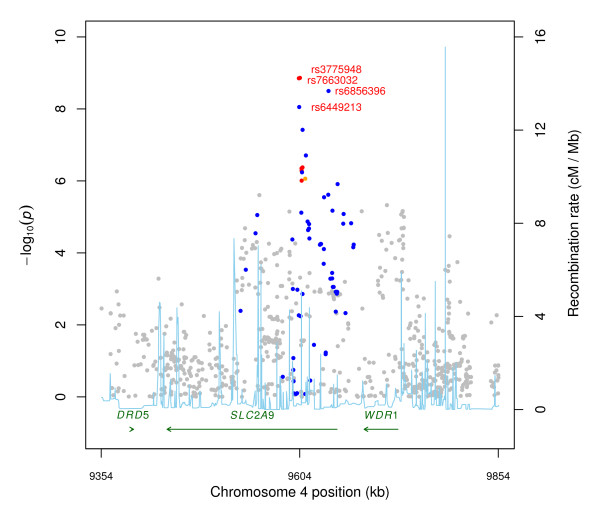
**Local genetic architecture**. Association *p*-values from multiple linear regression are shown based on physical position (NCBI build 36, dbSNP build 126). The light blue curve depicts the recombination rate from the combined Hap Map Phase II data. Linkage disequilibrium based on the HUFS sample is color-coded red for *r*^2 ^to the top SNP ≥ 0.8, orange for *r*^2 ^< 0.8 and ≥ 0.5, blue for *r*^2 ^< 0.5 and ≥ 0.2, and gray for *r*^2 ^< 0.20. Green arrows indicate the direction of transcription.

The four genome-wide significant associations were for SNPs rs3775948, rs7663032, rs6856396, and rs6449213 (Additional File 4). These 4 SNPs were among the eleven most frequently reported SNPs in *SLC2A9 *associated with serum uric acid in European-ancestry populations (Additional File 5) [16,34-40]. These four SNPs are all located in a linkage disequilibrium (LD) block on chromosome 4 (Figure 2) in the gene *SLC2A9 *(GeneID 56606). The next 8 highest ranking SNPs were also located in *SLC2A9 *(Additional File 4). The association at rs3775948 explained 2.6% of the phenotypic variance in our sample.

Serum uric acid levels were significantly different between males and females (Wilcoxon rank sum test, *p *= 4.55 × 10^-35^), with males having a mean serum uric acid level of 6.25 mg/dl compared to 5.02 mg/dl for females. The effect size estimates between females and males at rs6449213 trended toward being significantly different (Welch's *t*-test, *p *= 0.068). The top 25 ranked SNPs for serum uric acid in males and females, unadjusted for covariates, are provided in Additional Files 6 and 7, respectively. The top 25 ranked SNPs for serum uric acid, adjusted for age, BMI, hypertension, eGFR, T2D, and the top two PCs, in males and females are provided in Additional Files 8 and 9, respectively.

### Fine Mapping

**Figure 3:**
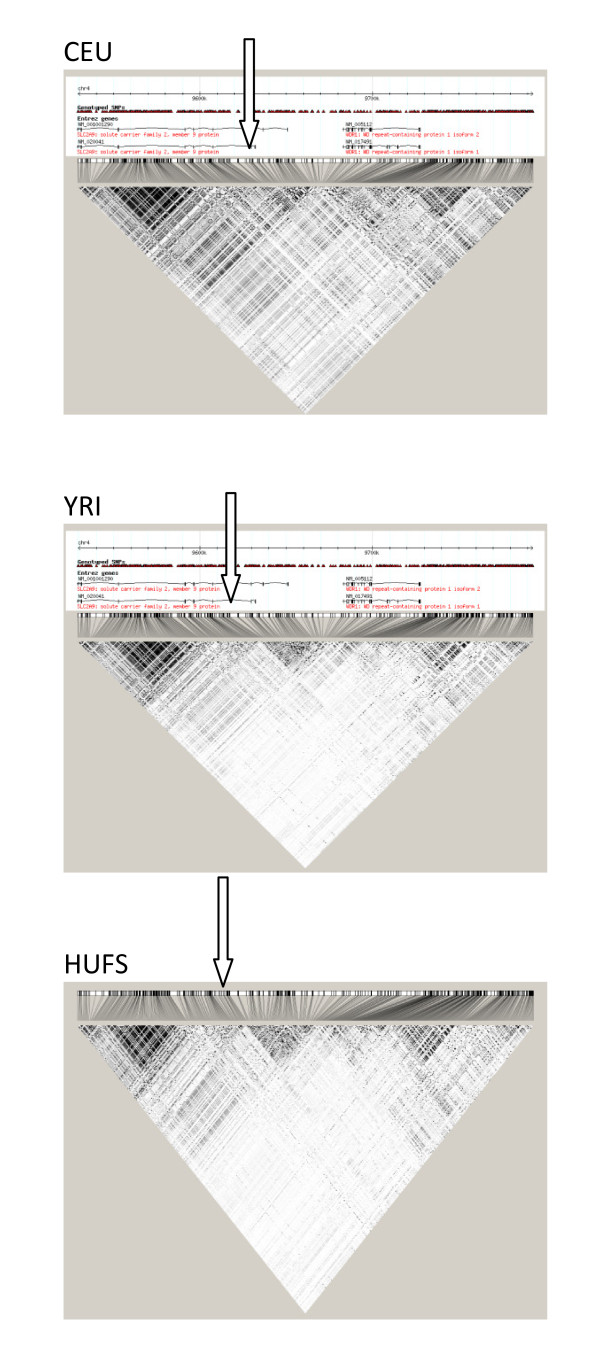
**Comparison of linkage disequilibrium in the HapMap Phase II CEU and YRI data and our HUFS sample**.

Using an *r*^2 ^cutoff of ≥ 0.3, LD with rs6449123 extends across an interval of approximately 263 kb in the CEU population and 231 kb in the YRI population. Restricting the *r*^2 ^cutoff to ≥0.5, LD with rs6449213 in the CEU population remains unchanged while, in sharp contrast, the LD interval in YRI is reduced to approximately 37 kb. All of the 10 top ranking SNPs fall within the 263 kb range; on the other hand, and quite remarkably 9 of the 10 top ranked SNPs lie within the 37 kb interval and 3 out of the 4 SNPs that achieved genome-wide significance lie in an approximately 1.3 kb interval (Figure 3).

## Discussion

Our findings replicate those of other investigators who found association between variants in *SLC2A9 *and SUAL [4,17,18,38]. To our knowledge, our study is the first to report association between *SLC2A9 *and uric acid in a large sample of admixed African Americans. The high expression of SLC2A9 in the epithelial cells of the proximal tubule and the atypical membranes of the kidneys [41], along with evidence that SLC2A9 is responsible for transport/re-absorption of uric acid and to a lesser extent glucose and fructose [42], provides biological plausibility for the high *p*-values for the SNPs in this gene in association with uric acid levels.

Hyperuricemia has been implicated in multiple physiologic outcomes including hypertension and renal dysfunction. Hyperuricemia is suspected to influence the development of hypertension via its role in vascular endothelial cell dysfunction and activation of the renin-angiotensin system [10]. Furthermore, experimental models demonstrating the causative effects of hyperuricemia in the development of hypertension were produced in rats with oxonic acid-induced hyperuricemia. These rats developed salt-resistant hypertension after induction of hyperuricemia, which resolved following reduction of uric acid to normal levels [43].

Hyperuricemia has also been demonstrated to increase the odds of developing acute renal dysfunction after cardiovascular surgery and increased the odds of developing chronic renal disease 4-fold and 3-fold, respectively, [44,45]. This information, coupled with evidence that hyperuricemia causes epithelial dysfunction in renal vessels [46], also supports the association we found between higher uric acid levels and reduced eGFR. The SNP rs6449213 has not only been associated with uric acid levels but this association was demonstrated to be influenced by sex and BMI [36], which may help explain the associations we found between uric acid, sex, and BMI in this study.

The findings of our fine-mapping analysis demonstrate the advantage of using African-ancestry populations in follow-up analyses of GWAS signals originally discovered in European-ancestry populations. Replication analysis, using follow-up samples of increasing numbers of individuals with European ancestry (more specifically, populations with similar linkage disequilibrium patterns), allows for assessment of replication and refinement of effect size estimates. In contrast, using follow-up samples of individuals with ancestry differing from the discovery sample (specifically, populations with weaker linkage disequilibrium patterns) potentially allows for resolution of the location of the GWAS signals through the weaker linkage disequilibrium patterns in the follow-up population. Capitalizing on the weaker linkage disequilibrium in African Americans compared to EA populations, we were able to fine-map the *SLC2A9 *signal from 263 kb to 37 kb.

Serum uric acid's association with sex is confirmed in our study. The association between decreased serum uric acid and the effect allele of rs6449213 in *SLC2A9 *was sex-specific, replicating the findings of other investigators [36,37]. Specifically, rs6449213 reached genome-wide significance in females but not in males. Given that rs6449213 has a smaller effect on serum uric acid in males than in females and that sex-stratified analysis reduces sample size, it is possible that the lack of association of rs6449213 with serum uric acid in males and the marginal *p*-value for the test of effect size estimates in females *vs*. males in our study both reflect false negatives finding.

A major limitation of our study is the moderate sample size compared to other GWAS studies. This suggests that loci with small effects may have been missed. The paucity of GWAS data on large numbers of African-Americans limits our ability to replicate our findings in a population with a similar substructure at this time. Despite these limitations, it is noteworthy that we replicated several of the reported association variants in the gene, *SLC2A9*. The effect size of this association (2.6% of the phenotypic variance averaged across sexes) is large compared to those from GWAS in general [40] but comparable to estimates from several studies of individuals of European ancestry [16,35,38,39].

## Conclusions

We found that *SLC2A9 *was significantly associated with serum uric acid in this population-based sample of African Americans, with a stronger effect in females than in males. Additionally, we observed significant association between uric acid levels and BMI, sex, eGFR, and hypertension. These observations deserve more in-depth evaluation in other human populations.

## Abbreviations

(*ABCG2*): Adenosine triphosphate-Binding Cassette, Subfamily G, Member 2; (AA): African American; (BMI): Body Mass index; (CEU): CEPH Utah residents with ancestry from northern and western Europe; (GWAS): Genome-wide Association Study; (GFR): Glomerular Filtration Rate; (*GCKR*): Glucokinase Regulatory Protein; (Hap Map): International Hap Map Consortium; (HWE): Hardy-Weinberg equilibrium; (HUFS): Howard University Family Study; (HTN): Hypertension; (*LRRC16A*): Leucine-Rich Repeat-Containing Protein 16A; (*PDZK1*): PDZ Domain-Containing Protein 1; (PC): Principal component; (*SLC2A9*): Solute Carrier Family 2, Member 9; (*SLC16A9*): Solute Carrier Family 16, Member 9,; (*SLC22A11*): Solute Carrier Family 22, Member 11; (*SLC2A12*): Solute Carrier Family 22, Member 12; (T2D): Type 2 Diabetes; (*WDR1*): WD repeat domain 1; (YRI): Yoruba in Ibadan, Nigeria

## Competing interests

The authors declare that they have no competing interests.

## Authors' contributions

Conception: AA, AH, MC, CR. Study design: BC, DS, AD, GC, JZ, HH, AH, NP, MC, AA, CR Sample processing and data management: HH, AD, NG, ZH. Genotyping: AD, AH, NG, HH, MC. Imputation: DS, AA, GC, AH. Analyzed the Data: BC, DS, AH, JZ, GC, NG. Interpretation: BC, DS, AD, AA, GC, CR, Drafted the manuscript: BC, DS. Revised manuscript: AA, CR. All authors read and approved the final manuscript.

## Pre-publication history

The pre-publication history for this paper can be accessed here:

http://www.biomedcentral.com/1755-8794/4/17/prepub

## Supplementary Material

Additional file 1 **Supplementary Figure S1**. Quantile-quantile plot for genomic control. The red line indicates the expected distribution. The inflation factor (λ_GC_) is shown. Click here for file

Additional file 2 **Supplementary Table S1**. Top 25 SNPs for serum uric acid, unadjusted for covariates. Click here for file

Additional file 3 **Supplementary Table S2**. Top 25 SNPs for serum uric acid, adjusted for age and sex. Click here for file

Additional file 4 **Table 3**. Top 25 SNPs for serum uric acid, adjusted for age, sex, BMI, HTN, eGFR, T2D, and the top two PCs. Click here for file

Additional file 5 **Table 4**. Previously reported GWAS associations between specific SNPs and serum uric acid levels. Click here for file

Additional file 6 **Supplementary Table S3**. Top 25 SNPs for serum uric acid in males, unadjusted for covariates. Click here for file

Additional file 7 **Supplementary Table S4**. Top 25 SNPs for serum uric acid in females, unadjusted for covariates. Click here for file

Additional file 8 **Supplementary Table S5**. Top 25 SNPs for serum uric acid in males, adjusted for age, BMI, HTN, eGFR, T2D, and the top two PCs. Click here for file

Additional file 9 **Supplementary Table S6**. Top 25 SNPs for serum uric acid in females, adjusted for age, BMI, HTN, eGFR, T2D, and the top two PCs. Click here for file
