# Supplementary figures and images for: A genome-wide association study of serum uric acid in African Americans
Source: BMC Med Genomics. 2011 Feb 4;4:17. doi: 10.1186/1755-8794-4-17 (PMC3045279; doi:10.1186/1755-8794-4-17)

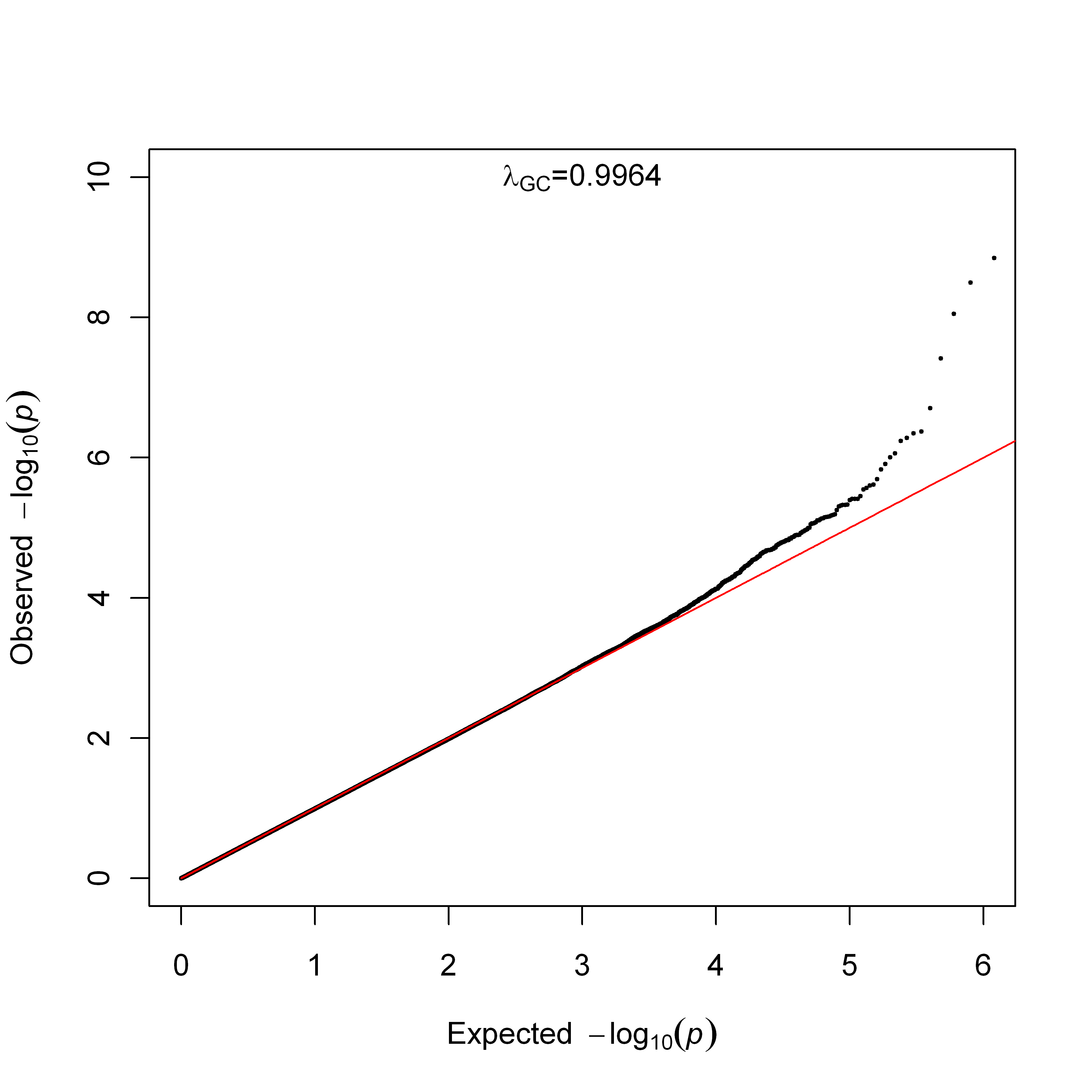

Supplement: Additional file 1 — Supplementary Figure S1. Quantile-quantile plot for genomic control. The red line indicates the expected distribution. The inflation factor (λGC) is shown. [file 1755-8794-4-17-S1.PNG]
