# Supplementary material for: A genome-wide association study of serum uric acid in African Americans
Source: BMC Med Genomics. 2011 Feb 4;4:17. doi: 10.1186/1755-8794-4-17 (PMC3045279; doi:10.1186/1755-8794-4-17)
Supplement: Additional file 2 — Supplementary Table S1. Top 25 SNPs for serum uric acid, unadjusted for covariates. [file 1755-8794-4-17-S2.DOC]

Supplementary Table S1: Top 25 SNPs for serum uric acid, unadjusted for covariates

| **SNP** | **Chr** | **Coordinate (bp)** | **Type** | **Closest Gene** | **Distance to Gene (bp)** | **Effect Allele** | **Effect Allele Frequency** | **β (SE)** | ***P*-value** |
| --- | --- | --- | --- | --- | --- | --- | --- | --- | --- |
| rs3775948 | 4 | 9,604,280 | Intronic | *SLC2A9* | 0 | G | 0.34 | -0.211 (0.037) | 1.00×10^-8^ |
| rs7663032 | 4 | 9,602,936 | Intronic | *SLC2A9* | 0 | C | 0.34 | -0.211 (0.037) | 1.09×10^-8^ |
| rs13113918 | 4 | 9,607,591 | Synonymous | *SLC2A9* | 0 | A | 0.23 | -0.218 (0.040) | 8.13×10^-8^ |
| rs4529048 | 4 | 9,606,210 | Intronic | *SLC2A9* | 0 | C | 0.32 | -0.189 (0.037) | 3.04×10^-7^ |
| rs10939650 | 4 | 9,607,538 | Synonymous | *SLC2A9* | 0 | C | 0.32 | -0.187 (0.037) | 3.89×10^-7^ |
| rs3733588 | 4 | 9,606,401 | Intronic | *SLC2A9* | 0 | G | 0.33 | -0.181 (0.036) | 7.16×10^-7^ |
| rs6449213 | 4 | 9,603,313 | Intronic | *SLC2A9* | 0 | C | 0.15 | -0.235 (0.047) | 8.38×10^-7^ |
| rs9991278 | 4 | 9,611,763 | Intronic | *SLC2A9* | 0 | T | 0.21 | -0.202 (0.041) | 1.21×10^-6^ |
| rs17111396 | 14 | 80,593,807 | Intronic | *TSHR* | 0 | A | 0.27 | -0.192 (0.040) | 1.35×10^-6^ |
| rs7669607 | 4 | 9,606,899 | Intronic | *SLC2A9* | 0 | T | 0.18 | -0.213 (0.044) | 1.79×10^-6^ |
| rs7517537 | 4 | 148,380,707 | Intronic | *VPS45* | 0 | T | 0.14 | -0.227 (0.047) | 1.80×10^-6^ |
| rs6549373 | 3 | 66,852,574 | Intergenic | *RP11-814M22.1* | 82,048 | T | 0.06 | -0.349 (0.073) | 2.01×10^-6^ |
| rs12058524 | 1 | 148,333,008 | Intronic | *VPS45* | 0 | T | 0.15 | -0.223 (0.047) | 2.03×10^-6^ |
| rs1014290 | 4 | 9,610,959 | Intronic | *SLC2A9* | 0 | G | 0.31 | -0.176 (0.037) | 2.41×10^-6^ |
| rs1061377 | 4 | 38,801,145 | Intronic | *RP11-360F5.1* | 0 | A | 0.46 | 0.160 (0.034) | 3.06×10^-6^ |
| rs7675782 | 4 | 38,798,063 | Intronic | *RP11-360F5.1* | 0 | A | 0.46 | 0.159 (0.034) | 3.31×10^-6^ |
| rs12192947 | 6 | 24,426,549 | Intronic | *DCDC2* | 0 | G | 0.26 | -0.176 (0.038) | 4.56×10^-6^ |
| rs3788483 | 22 | 31,414,345 | Intronic | *Z73979.2* | 0 | C | 0.31 | 0.164 (0.036) | 5.42×10^-6^ |
| rs2024424 | 14 | 80,590,132 | Intronic | *TSHR* | 0 | C | 0.23 | -0.184 (0.040) | 5.56×10^-6^ |
| rs10456303 | 6 | 24,425,378 | Intronic | *DCDC2* | 0 | T | 0.26 | -0.174 (0.038) | 6.18×10^-6^ |
| rs2737618 | 1 | 98,374,048 | Intronic | *NR5A2* | 0 | A | 0.07 | 0.311 (0.069) | 6.68×10^-6^ |
| rs6856396 | 4 | 9,640,261 | Intronic | *SLC2A9* | 0 | A | 0.19 | -0.198 (0.044) | 7.47×10^-6^ |
| rs17159991 | 7 | 3,120,995 | Within noncoding | *AC006978.1* | 0 | A | 0.05 | -0.329 (0.073) | 7.67×10^-6^ |
| rs16875249 | 6 | 14,690,423 | Intergenic | *RP11-330A16.1* | 15,301 | A | 0.10 | 0.176 (0.039) | 7.75×10^-6^ |
| rs6871803 | 5 | 62,047,739 | Intergenic | *ISCA1P1* | 59,219 | T | 0.24 | 0.176 (0.039) | 8.00×10^-6^ |
