# Supplementary material for: A genome-wide association study of serum uric acid in African Americans
Source: BMC Med Genomics. 2011 Feb 4;4:17. doi: 10.1186/1755-8794-4-17 (PMC3045279; doi:10.1186/1755-8794-4-17)
Supplement: Additional file 3 — Supplementary Table S2. Top 25 SNPs for serum uric acid, adjusted for age and sex. [file 1755-8794-4-17-S3.DOC]

Supplementary Table S2: Top 25 SNPs for serum uric acid, adjusted for age and sex

| **SNP** | **Chr** | **Coordinate (bp)** | **Type** | **Closest Gene** | **Distance to Gene (bp)** | **Effect Allele** | **Effect Allele Frequency** | **β (SE)** | ***P-*value** |
| --- | --- | --- | --- | --- | --- | --- | --- | --- | --- |
| rs7663032 | 4 | 9,602,936 | Intronic | *SLC2A9* | 0 | C | 0.34 | -0.210 (0.033) | 3.74×10^-10^ |
| rs3775948 | 4 | 9,604,280 | Intronic | *SLC2A9* | 0 | G | 0.34 | -0.210 (0.033) | 3.74×10^-10^ |
| rs13113918 | 4 | 9,607,591 | Synonymous | *SLC2A9* | 0 | A | 0.23 | -0.210 (0.037) | 1.26×10^-8^ |
| rs4529048 | 4 | 9,606,210 | Intronic | *SLC2A9* | 0 | C | 0.32 | -0.184 (0.033) | 3.60×10^-8^ |
| rs10939650 | 4 | 9,607,538 | Synonymous | *SLC2A9* | 0 | C | 0.32 | -0.182 (0.033) | 5.03×10^-8^ |
| rs3733588 | 4 | 9,606,401 | Intronic | *SLC2A9* | 0 | G | 0.33 | -0.178(0.033) | 7.98×10^-8^ |
| rs2573674 | 15 | 98,294,206 | Intergenic | *ADAMTS17* | 35,111 | A | 0.38 | -0.170 (0.032) | 1.39×10^-7^ |
| rs9991278 | 4 | 9,611,763 | Intronic | *SLC2A9* | 0 | T | 0.21 | -0.195 (0.038) | 2.53×10^-7^ |
| rs6449213 | 4 | 9,603,313 | Intronic | *SLC2A9* | 0 | C | 0.15 | -0.221 (0.043) | 3.08×10^-7^ |
| rs6856396 | 4 | 9,640,261 | Intronic | *SLC2A9* | 0 | A | 0.19 | -0.202 (0.040) | 4.50×10^-7^ |
| rs1014290 | 4 | 9,610,959 | Intronic | *SLC2A9* | 0 | G | 0.31 | -0.170 (0.034) | 4.74×10^-7^ |
| rs2581360 | 15 | 98,309,405 | Intergenic | *ADAMTS17* | 19,912 | T | 0.38 | -0.160 (0.032) | 6.32×10^-7^ |
| rs2244967 | 10 | 49,894,772 | Within noncoding | *RP11-523O18.1* | 0 | T | 0.46 | -0.149 (0.030) | 1.02×10^-6^ |
| rs2254410 | 10 | 49,908,361 | Intronic | *C10orf72* | 0 | G | 0.47 | -0.147 (0.031) | 1.74×10^-6^ |
| rs7669607 | 4 | 9,606,899 | Intronic | *SLC2A9* | 0 | T | 0.18 | -0.194 (0.040) | 1.79×10^-6^ |
| rs1347477 | 15 | 98,321,229 | Intergenic | *ADAMTS17* | 19,088 | G | 0.41 | -0.147 (0.031) | 2.72×10^-6^ |
| rs17111396 | 14 | 80,593,807 | Intronic | *TSHR* | 0 | A | 0.27 | -0.169 (0.036) | 2.84×10^-6^ |
| rs11036476 | 11 | 5,231,919 | Intronic | *HBG2* | 0 | T | 0.23 | -0.170 (0.036) | 3.08×10^-6^ |
| rs9965063 | 18 | 64,759,618 | Intronic | *CCDC102B* | 0 | A | 0.04 | 0.375 (0.080) | 3.26×10^-6^ |
| rs356128 | 9 | 96,611,051 | Intronic | *C9orf3* | 0 | C | 0.05 | -0.309 (0.066) | 3.47×10^-6^ |
| rs7855781 | 9 | 96,618,259 | Intronic | *C9orf3* | 0 | A | 0.05 | -0.309 (0.066) | 3.47×10^-6^ |
| rs894425 | 9 | 96,624,838 | Intronic | *C9orf3* | 0 | T | 0.05 | -0.309 (0.066) | 3.47×10^-6^ |
| rs3887267 | 9 | 96,539,005 | Intronic | *C9orf3* | 0 | T | 0.05 | -0.309 (0.066) | 3.51×10^-6^ |
| rs60120 | 9 | 96,564,924 | Intronic | *C9orf3* | 0 | C | 0.05 | -0.309 (0.066) | 3.51×10^-6^ |
| rs438742 | 9 | 96,572,185 | Intronic | *C9orf3* | 0 | G | 0.05 | -0.309 (0.066) | 3.51×10^-6^ |
