# Supplementary material for: A genome-wide association study of serum uric acid in African Americans
Source: BMC Med Genomics. 2011 Feb 4;4:17. doi: 10.1186/1755-8794-4-17 (PMC3045279; doi:10.1186/1755-8794-4-17)
Supplement: Additional file 4 — Table 3. Top 25 SNPs for serum uric acid, adjusted for age, sex, BMI, HTN, eGFR, T2D, and the top two PCs. [file 1755-8794-4-17-S4.DOC]

**Table 3:** Top 25 SNPs for serum uric acid, adjusted for age, sex, BMI, HTN, eGFR, T2D, and the top two PCs

| **SNP** | **Chr** | **Coordinate**  **(bp)** | **Type** | **Closest Gene** | **Distance to Gene (bp)** | **Effect Allele** | **Effect Allele Frequency** | **β (SE)** | ***P*-value** |
| --- | --- | --- | --- | --- | --- | --- | --- | --- | --- |
| rs3775948 | 4 | 9,604,280 | Intronic | *SLC2A9* | 0 | G | 0.34 | -0.183 (0.030) | 1.38×10^-9^ |
| rs7663032 | 4 | 9,602,936 | Intronic | *SLC2A9* | 0 | C | 0.34 | -0.183 (0.030) | 1.41×10^-9^ |
| rs6856396 | 4 | 9,640,261 | Intronic | *SLC2A9* | 0 | A | 0.19 | -0.213 (0.036) | 3.18×10^-9^ |
| rs6449213 | 4 | 9,603,313 | Intronic | *SLC2A9* | 0 | C | 0.15 | -0.224 (0.039) | 8.88×10^-9^ |
| rs13113918 | 4 | 9,607,591 | Synonymous | *SLC2A9* | 0 | A | 0.24 | -0.184 (0.033) | 3.82×10^-8^ |
| rs9991278 | 4 | 9,611,763 | Synonymous | *SLC2A9* | 0 | T | 0.32 | -0.177 (0.034) | 1.97×10^-7^ |
| rs10939650 | 4 | 9,607,538 | Synonymous | *SLC2A9* | 0 | C | 0.32 | -0.153 (0.030) | 4.23×10^-7^ |
| rs4529048 | 4 | 9,606,210 | Intronic | *SLC2A9* | 0 | C | 0.32 | -0.153 (0.030) | 4.49×10^-7^ |
| rs7669607 | 4 | 9,606,800 | Intronic | *SLC2A9* | 0 | T | 0.18 | -0.183 (0.036) | 5.78×10^-7^ |
| rs1014290 | 4 | 9,610,959 | Intronic | *SLC2A9* | 0 | G | 0.31 | -0.150 (0.030) | 8.68×10^-7^ |
| rs3733588 | 4 | 9,606,401 | Intronic | *SLC2A9* | 0 | G | 0.33 | -0.147 (0.030) | 9.86×10^-7^ |
| rs7349721 | 4 | 9,651,660 | Intronic | *SLC2A9* | 0 | A | 0.22 | -0.163 (0.033) | 1.23×10^-6^ |
| rs6942328 | 6 | 4,457,978 | Intergenic | *AL162718.1* | 84,664 | C | 0.02 | 0.545 (0.112) | 1.47×10^-6^ |
| rs9480441 | 6 | 157,480,953 | Intronic | *SLC2A9* | 0 | A | 0.28 | -0.147 (0.031) | 2.02×10^-6^ |
| rs6826764 | 4 | 9,639,892 | Intronic | *SLC2A9* | 0 | G | 0.22 | -0.158 (0.033) | 2.48×10^-6^ |
| rs12498742 | 4 | 9,553,150 | Intronic | *SLC2A9* | 0 | A | 0.48 | 0.134 (0.028) | 1.94×10^-6^ |
| rs9478751 | 6 | 157,482,742 | Intronic | *ARID1B* | 0 | A | 0.30 | 0.145 (0.031) | 2.70×10^-6^ |
| rs3756231 | 4 | 9,634,642 | Intronic | *SLC2A9* | 0 | G | 0.12 | -0.200 (0.043) | 2.84×10^-6^ |
| rs8139900 | 22 | 24,724,724 | Intronic | *MYO18B* | 0 | A | 0.21 | 0.169 (0.036) | 3.51×10^-6^ |
| rs2047267 | 5 | 25,123,482 | Within noncoding | *AC106810.1* | 0 | G | 0.17 | 0.170 (0.037) | 3.86×10^-6^ |
| rs6452245 | 5 | 25,123,886 | Within noncoding | *AC106810.1* | 0 | G | 0.36 | -0.170 (0.037) | 3.86×10^-6^ |
| rs10059755 | 5 | 25,124,598 | Within noncoding | *AC106810.1* | 0 | A | 0.17 | 0.170 (0.037) | 3.86×10^-6^ |
| rs1982821 | 3 | 179,275,801 | Intergenic | *AC007953.1* | -67,540 | T | 0.17 | -0.171 (0.037) | 4.02×10^-6^ |
| rs2244967 | 10 | 49,894,772 | Within noncoding | *RP11-523018.1* | 0 | T | 0.46 | -0.126 (0.027) | 4.67×10^-6^ |
| rs717615 | 4 | 9,713,768 | Intronic | *WDR1* | 0 | G | 0.35 | -0.130 (0.028) | 4.72×10^-6^ |
