# Supplementary material for: A genome-wide association study of serum uric acid in African Americans
Source: BMC Med Genomics. 2011 Feb 4;4:17. doi: 10.1186/1755-8794-4-17 (PMC3045279; doi:10.1186/1755-8794-4-17)
Supplement: Additional file 5 — Table 4. Previously reported GWAS associations between specific SNPs and serum uric acid levels. [file 1755-8794-4-17-S5.DOC]

**Table 4:** Previously reported associations between specific SNPs in *SLC2A9* and serum uric acid levels

| **SNP** | **Chromosome** | **Effect Allele** | **Other Allele** | **Sample Size** | **β** | **SE** | ***P*-value** | **Reference** | **Study** |
| --- | --- | --- | --- | --- | --- | --- | --- | --- | --- |
| rs1014290 | 4 | G | A | 944 | -0.320 | 0.046 | 7.50×10^-12^ | [35] | CROATIA |
| rs12498742 | 4 | A | G | 28,035 | 0.308 | 0.010 | 1.98×10^-200^ | [16] | Meta-Analysis |
| rs13111638 | 4 | C | T | 868 | 0.450 | 0.070 | 4.04×10^-10^ | [38] | HAPI |
| rs3733588 | 4 | C | T | 868 | -0.290 | 0.060 | 9.30×10^-6^ | [38] | HAPI |
| rs3775948 | 4 | C | G | 27,968 | 0.283 | 0.010 | 4.23×10^-180^ | [16] | Meta-Analysis |
| rs4320137 | 4 | T | C | 27,792 | 0.283 | 0.013 | 3.02×10^-113^ | [16] | Meta-Analysis |
|  | 4 | C | T | 868 | 0.470 | 0.080 | 7.83×10^-10^ | [38] | HAPI |
| rs4529048 | 4 | A | C | 868 | 0.300 | 0.060 | 1.64×10^-6^ | [38] | HAPI |
|  | 4 | A | C | 27,958 | 0.279 | 0.010 | 4.15×10^-175^ | [16] | Meta-Analysis |
| rs6449213 | 4 | C | T | 868 | -0.470 | 0.070 | 3.36×10^-10^ | [38] | HAPI |
|  | 4 | C | T | 2,669 | -0.357 | NA | 2.33×10^-11^ | [36] | Bruneck |
|  | 4 | C | T | 7,699 | -0.370 | 0.020 | 2.90×10^-68^ | [34] | FHS |
|  | 4 | C | T | 4,148 | -0.320 | 0.030 | 1.15×10^-29^ | [34] | Rotterdam |
|  | 4 | C | T | 11,847 | -0.350 | 0.020 | 2.20×10^-104^ | [34] | All White |
|  | 4 | G | A | 944 | -0.330 | 0.054 | 1.20×10^-9^ | [35] | CROATIA |
|  | 4 | T | C | 28,006 | 0.323 | 0.011 | 4.79×10^-186^ | [16] | Meta-Analysis |
| rs6826764 | 4 | C | G | 28,044 | 0.250 | 0.011 | 1.26×10^-110^ | [16] | Meta-Analysis |
|  | 4 | C | G | 868 | -0.390 | 0.070 | 4.27×10^-8^ | [38] | HAPI |
| rs7663032 | 4 | T | C | 14,554 | 0.270 | 0.013 | 3.34×10^-97^ | [16] | Meta-Analysis |
| rs733175 | 4 | G | A | 944 | -0.252 | 0.052 | 1.80×10^-6^ | [35] | CROATIA |
