# Supplementary material for: A genome-wide association study of serum uric acid in African Americans
Source: BMC Med Genomics. 2011 Feb 4;4:17. doi: 10.1186/1755-8794-4-17 (PMC3045279; doi:10.1186/1755-8794-4-17)
Supplement: Additional file 6 — Supplementary Table S3. Top 25 SNPs for serum uric acid in males, unadjusted for covariates. [file 1755-8794-4-17-S6.DOC]

Supplementary Table S3: Top 25 SNPs for serum uric acid in males, unadjusted for covariates

| **SNP** | **Chr** | **Coordinate (bp)** | **Type** | **Closest Gene** | **Distance to Gene (bp)** | **Effect Allele** | **Effect Allele Frequency** | **β (SE)** | ***P-*value** |
| --- | --- | --- | --- | --- | --- | --- | --- | --- | --- |
| rs1484022 | 4 | 173,452,366 | Intronic | *GALNTL6* | 0 | A | 0.12 | 0.322 (0.068) | 2.81×10^-6^ |
| rs2626622 | 4 | 173,460,458 | Intronic | *GALNTL6* | 0 | T | 0.12 | 0.321 (0.068) | 2.81×10^-6^ |
| rs2626612 | 4 | 173,451,296 | Intronic | *GALNTL6* | 0 | T | 0.18 | 0.059 (0.164) | 3.16×10^-6^ |
| rs2626613 | 4 | 173,451,354 | Intronic | *GALNTL6* | 0 | A | 0.18 | 0.281 (0.164) | 3.16×10-^6^ |
| rs9997921 | 4 | 173,453,448 | Intronic | *GALNTL6* | 0 | C | 0.18 | 0.281 (0.059) | 3.16×10^-6^ |
| rs166238 | 5 | 123,200,965 | Intergenic | *AC008541.1* | 182,055 | C | 0.28 | 0.237 (0.050) | 3.38×10^-6^ |
| rs6553622 | 4 | 173,453,802 | Intronic | *GALNTL6* | 0 | C | 0.18 | 0.279 (0.060) | 3.90×10^-6^ |
| rs2961923 | 5 | 159,727,089 | Intronic | *C1QTNF2* | 0 | A | 0.30 | 0.235 (0.051) | 4.98×10^-6^ |
| rs8036863 | 15 | 92,543,003 | Intergenic | *MCTP2* | -31,952 | A | 0.14 | -0.303 (0.066) | 5.63×10^-6^ |
| rs153877 | 5 | 123,199,772 | Intergenic | *AC008541.1* | 180,862 | C | 0.27 | 0.231 (0.051) | 6.77×10^-6^ |
| rs6490765 | 13 | 22,473,968 | Intergenic | *AL157931.1* | -23,832 | G | 0.44 | -0.216 (0.047) | 6.82×10^-6^ |
| rs9487680 | 6 | 112,033,541 | Intronic | *TRAF3IP2* | 0 | G | 0.13 | 0.293 (0.064) | 6.95×10^-6^ |
| rs9638655 | 7 | 71,475,237 | Intronic | *CALN1* | 0 | A | 0.45 | -0.221 (0.049) | 7.00×10^-6^ |
| rs9325825 | 8 | 17,998,088 | Within noncoding | *AC124242.2* | 0 | T | 0.15 | -0.292 (0.064) | 7.30×10^-6^ |
| rs12153391 | 5 | 171,136,043 | Intergenic | *C5orf50* | -9,438 | A | 0.13 | -0.331 (0.073) | 8.13×10^-6^ |
| rs3783412 | 14 | 49,926,391 | Intronic | *CDKL1* | 0 | A | 0.32 | 0.222 (0.049) | 8.66×10^-6^ |
| rs9590754 | 13 | 42,763,812 | Intronic | *ENOX1* | 0 | T | 0.07 | -0.413 (0.092) | 9.20×10^-6^ |
| rs11130076 | 3 | 45,828,076 | Intergenic | *LZTFL1* | 11,736 | T | 0.12 | 0.312 (0.069) | 9.36×10^-6^ |
| rs3910245 | 11 | 5,550,043 | Intronic | *HBG2* | 0 | A | 0.23 | -0.240(0.054) | 9.75×10^-6^ |
| rs556356 | 11 | 64,260,072 | Intronic | *RASGRP2* | 0 | A | 0.10 | 0.343 (0.077) | 9.86×10^-6^ |
| rs2138193 | 2 | 180,215,179 | Intronic | *ZNF385B* | 0 | A | 0.10 | 0.311 (0.070) | 1.05×10^-5^ |
| rs6995047 | 8 | 18,023,461 | Intergenic | *AC124242.2* | 24,055 | A | 0.15 | -0.279 (0.063) | 1.08×10^-5^ |
| rs534221 | 11 | 64,253,737 | Intronic | *RASGRP2* | 0 | C | 0.10 | 0.340 (0.077) | 1.26×10^-5^ |
| rs4628288 | 8 | 17,995,037 | Within noncoding | *AC124242.2* | 0 | T | 0.14 | -0.292 (0.066) | 1.32×10^-5^ |
| rs7015927 | 8 | 17,999,419 | Downstream | *AC124242.2* | 13 | A | 0.15 | -0.285 (0.065) | 1.35×10^-5^ |
