# Supplementary material for: A genome-wide association study of serum uric acid in African Americans
Source: BMC Med Genomics. 2011 Feb 4;4:17. doi: 10.1186/1755-8794-4-17 (PMC3045279; doi:10.1186/1755-8794-4-17)
Supplement: Additional file 7 — Supplementary Table S4. Top 25 SNPs for serum uric acid in females, unadjusted for covariates. [file 1755-8794-4-17-S7.DOC]

Supplementary Table S4: Top 25 SNPs for serum uric acid in females, unadjusted for covariates

| **SNP** | **Chr** | **Coordinate (bp)** | **Type** | **Closest Gene** | **Distance to Gene (bp)** | **Effect Allele** | **Effect Allele Frequency** | **β (SE)** | ***P-*value** |
| --- | --- | --- | --- | --- | --- | --- | --- | --- | --- |
| rs13113918 | 4 | 9,607,591 | Synonymous | *SLC2A9* | 0 | A | 0.76 | -0.270 (0.048) | 2.30×10^-8^ |
| rs3775948 | 4 | 9,604,280 | Intronic | *SLC2A9* | 0 | G | 0.34 | -0.237 (0.043) | 6.74×10^-8^ |
| rs7663032 | 4 | 9,602,936 | Intronic | *SLC2A9* | 0 | C | 0.34 | -0.237 (0.043) | 7.26×10^-8^ |
| rs9991278 | 4 | 9,611,763 | Intronic | *SLC2A9* | 0 | T | 0.21 | -0.254 (0.049) | 3.23×10^-7^ |
| rs7669607 | 4 | 9,606,899 | Intronic | *SLC2A9* | 0 | T | 0.18 | -0.266 (0.052) | 4.17×10^-7^ |
| rs6449213 | 4 | 9,603,313 | Intronic | *SLC2A9* | 0 | C | 0.15 | -0.280 (0.055) | 5.33×10^-7^ |
| rs4529048 | 4 | 9,606,210 | Intronic | *SLC2A9* | 0 | C | 0.32 | -0.218 (0.044) | 7.75×10^-7^ |
| rs10939650 | 4 | 9,607,538 | Synonymous | *SLC2A9* | 0 | C | 0.32 | -0.217 (0.044) | 8.89×10^-7^ |
| rs3733588 | 4 | 9,606,401 | Intronic | *SLC2A9* | 0 | G | 0.33 | -0.207 (0.044) | 2.35×10^-6^ |
| rs17155553 | 5 | 102,970,100 | Intergenic | *NUDT12* | -43,707 | T | 0.25 | 0.221 (0.047) | 2.95×10^-6^ |
| rs17087256 | 5 | 96,400,774 | Downstream | *LNPEP* | 1,799 | G | 0.10 | 0.322 (0.069) | 3.41×10^-6^ |
| rs1014290 | 4 | 9,610,959 | Intronic | *SLC2A9* | 0 | G | 0.31 | -0.207 (0.044) | 3.53×10^-6^ |
| rs10075546 | 5 | 102,972,611 | Intergenic | *NUDT12* | -46,218 | G | 0.25 | 0.218 (0.047) | 3.92×10^-6^ |
| rs17087274 | 5 | 96,404,925 | Intergenic | *LNPEP* | 5,950 | G | 0.10 | 0.317 (0.068) | 4.19×10^-6^ |
| rs10054393 | 5 | 102,986,669 | Intergenic | *NUDT12* | -60,276 | C | 0.33 | 0.202 (0.044) | 4.43×10^-6^ |
| rs717615 | 4 | 9,713,768 | Intronic | *WDR1* | 0 | G | 0.34 | -0.193 (0.042) | 4.95×10^-6^ |
| rs10738747 | 9 | 26,174,578 | Intergenic | *AL356791.1* | 66,172 | G | 0.39 | -0.199 (0.043) | 5.03×10^-6^ |
| rs3985863 | 15 | 45,820,660 | Intronic | *SEMA6D* | 0 | A | 0.03 | 0.540 (0.118) | 5.67×10^-6^ |
| rs987607 | 2 | 183,777,732 | Intergenic | *NUP35* | 43,079 | G | 0.27 | -0.200 (0.044) | 5.96×10^-6^ |
| rs7172576 | 15 | 29,342,618 | Intergenic | *KLF13* | -63,732 | G | 0.49 | -0.187 (0.041) | 5.99×10^-6^ |
| rs3887267 | 9 | 96,539,005 | Intronic | *C9orf3* | 0 | T | 0.05 | -0.395 (0.087) | 6.77×10^-6^ |
| rs60120 | 9 | 96,564,924 | Intronic | *C9orf3* | 0 | C | 0.05 | -0.395 (0.087) | 6.77×10^-6^ |
| rs438742 | 9 | 96,572,185 | Intronic | *C9orf3* | 0 | G | 0.05 | -0.395 (0.087) | 6.77×10^-6^ |
| rs562605 | 9 | 96,576,525 | Intronic | *C9orf3* | 0 | A | 0.05 | -0.395 (0.087) | 6.77×10^-6^ |
| rs356148 | 9 | 96,581,398 | Intronic | *C9orf3* | 0 | A | 0.05 | -0.395 (0.087) | 6.77×10^-6^ |
