# Supplementary material for: A genome-wide association study of serum uric acid in African Americans
Source: BMC Med Genomics. 2011 Feb 4;4:17. doi: 10.1186/1755-8794-4-17 (PMC3045279; doi:10.1186/1755-8794-4-17)
Supplement: Additional file 8 — Supplementary Table S5. Top 25 SNPs for serum uric acid in males, adjusted for age, BMI, HTN, eGFR, T2D, and the top two PCs. [file 1755-8794-4-17-S8.DOC]

Supplementary Table S5: Top 25 SNPs for serum uric acid in males, adjusted for age, BMI, HTN, eGFR, T2D, and the top two PCs

| **SNP** | **Chr** | **Coordinate (bp)** | **Type** | **Closest Gene** | **Distance to Gene (bp)** | **Effect Allele** | **Effect Allele Frequency** | **β (SE)** | ***P-*value** |
| --- | --- | --- | --- | --- | --- | --- | --- | --- | --- |
| rs9325825 | 8 | 17,998,088 | Within noncoding | *AC124242.2* | 0 | T | 0.15 | -0.301 (0.060) | 9.25×10^-7^ |
| rs6995047 | 8 | 18,023,461 | Intergenic | *AC124242.2* | 24,055 | A | 0.15 | -0.290 (0.059) | 1.26×10^-6^ |
| rs9638655 | 7 | 71,475,237 | Intronic | *CALN1* | 0 | A | 0.45 | -0.223 (0.046) | 1.50×10^-6^ |
| rs7015927 | 8 | 17,999,419 | Downstream | *AC124242.2* | 13 | A | 0.15 | -0.295 (0.061) | 1.66×10^-6^ |
| rs13280846 | 8 | 18,000,545 | Downstream | *AC124242.2* | 1,139 | A | 0.15 | -0.295 (0.061) | 1.66×10^-6^ |
| rs1999022 | 6 | 4,242,285 | Intergenic | *RP3527G5.1* | 48,691 | A | 0.27 | -0.241 (0.050) | 1.80×10^-6^ |
| rs4628288 | 8 | 17,995,037 | Within noncoding | *AC124242.2* | 0 | T | 0.14 | -0.300 (0.062) | 2.00×10^-6^ |
| rs6586693 | 8 | 17,995,285 | Within noncoding | *AC124242.2* | 0 | C | 0.14 | -0.299 (0.062) | 2.36×10^-6^ |
| rs6904215 | 6 | 4,228,691 | Intergenic | *RP11-625P7.1* | 56,187 | A | 0.30 | -0.218 (0.046) | 3.42×10^-6^ |
| rs2783030 | 6 | 107,451,052 | Intergenic | *C6orf203* | -5,048 | T | 0.25 | -0.228 (0.048) | 3.47×10^-6^ |
| rs6421410 | 8 | 18,002,533 | Downstream | *AC124242.2* | 3,127 | A | 0.15 | -0.278 (0.060) | 4.19×10^-6^ |
| rs11984007 | 7 | 37,557,232 | Intergenic | *AC009530.1* | 34,396 | A | 0.10 | 0.310 (0.067) | 4.78×10^-6^ |
| rs12413439 | 10 | 2,158,400 | Intergenic | *AL441943.1* | 27,937 | T | 0.03 | -0.566 (0.123) | 5.30×10^-6^ |
| rs12412835 | 10 | 2,159,025 | Intergenic | *AL441943.1* | 28,562 | C | 0.03 | -0.564 (0.123) | 5.98×10^-6^ |
| rs6586689 | 8 | 17,992,334 | Within noncoding | *AC124242.2* | 0 | C | 0.12 | -0.308 (0.067) | 6.04×10^-6^ |
| rs7148084 | 14 | 27,134,085 | Intergenic | *CTD-3006G17.1* | -335,873 | T | 0.13 | -0.302 (0.066) | 6.86×10^-6^ |
| rs1179375 | 3 | 55,718,238 | Intronic | *ERC2* | 0 | C | 0.46 | -0.202 (0.044) | 7.13×10^-6^ |
| rs938545 | 3 | 110,951,230 | Within noncoding | *RP11-457K10.1* | 0 | A | 0.03 | -0.732 (0.161) | 7.19×10^-6^ |
| rs918975 | 7 | 37,557,478 | Intergenic | *AC009530.1* | 34,150 | T | 0.10 | 0.306 (0.068) | 7.49×10^-6^ |
| rs9940636 | 16 | 54,486,422 | Intergenic | *CES7* | -19,639 | C | 0.07 | 0.399 (0.088) | 7.73×10^-6^ |
| rs17112910 | 14 | 27,138,492 | Intergenic | *CTD-3006G17.1* | -340,280 | G | 0.13 | -0.298 (0.066) | 8.01×10^-6^ |
| rs17112915 | 14 | 27,139,115 | Intergenic | *CTD-3006G17.1* | -340,903 | G | 0.13 | -0.298 (0.066) | 8.01×10^-6^ |
| rs2072012 | 22 | 24,571,994 | Intronic | *MYO18B* | 0 | C | 0.24 | -0.221 (0.049) | 8.23×10^-6^ |
| rs1954522 | 14 | 27,141,109 | Intergenic | *CTD-3006G17.1* | -342,897 | T | 0.13 | -0.298 (0.066) | 8.28×10^-6^ |
| rs7011695 | 8 | 18,015,839 | Intergenic | *AC124242.2* | 16,433 | T | 0.19 | -0.243 (0.054) | 8.43×10^-6^ |
