# Supplementary material for: A genome-wide association study of serum uric acid in African Americans
Source: BMC Med Genomics. 2011 Feb 4;4:17. doi: 10.1186/1755-8794-4-17 (PMC3045279; doi:10.1186/1755-8794-4-17)
Supplement: Additional file 9 — Supplementary Table S6. Top 25 SNPs for serum uric acid in females, adjusted for age, BMI, HTN, eGFR, T2D, and the top two PCs. [file 1755-8794-4-17-S9.DOC]

Supplementary Table S6: Top 25 SNPs for serum uric acid in females, adjusted for age, BMI, HTN, eGFR, T2D, and the top two PCs

| **SNP** | **Chr** | **Coordinate (bp)** | **Type** | **Closest Gene** | **Distance to Gene (bp)** | **Effect Allele** | **Effect Allele Frequency** | **β (SE)** | ***P-*value** |
| --- | --- | --- | --- | --- | --- | --- | --- | --- | --- |
| rs6449213 | 4 | 9,603,313 | Intronic | *SLC2A9* | 0 | C | 0.15 | -0.284 (0.048) | 6.46×10^-9^ |
| rs13113918 | 4 | 9,607,591 | Synonymous | *SLC2A9* | 0 | A | 0.23 | -0.235 (0.042) | 3.72×10^-8^ |
| rs7669607 | 4 | 9,606,899 | Intronic | *SLC2A9* | 0 | T | 0.18 | -0.249 (0.046) | 6.39×10^-8^ |
| rs3775948 | 4 | 9,604,280 | Intronic | *SLC2A9* | 0 | G | 0.34 | -0.208 (0.038) | 8.32×10^-8^ |
| rs7663032 | 4 | 9,602,936 | Intronic | *SLC2A9* | 0 | C | 0.34 | -0.207 (0.038) | 9.20×10^-8^ |
| rs6856396 | 4 | 9,640,261 | Intronic | *SLC2A9* | 0 | A | 0.19 | -0.248 (0.046) | 1.03×10^-7^ |
| rs9991278 | 4 | 9,611,763 | Intronic | *SLC2A9* | 0 | T | 0.21 | -0.230 (0.043) | 1.40×10^-7^ |
| rs717615 | 4 | 9,713,768 | Intronic | *WDR1* | 0 | G | 0.34 | -0.183 (0.037) | 8.19×10^-7^ |
| rs10054393 | 5 | 102,986,669 | Intergenic | *NUDT12* | -60,276 | C | 0.33 | 0.191 (0.038) | 8.74×10^-7^ |
| rs6915400 | 6 | 4,457,952 | Intergenic | *AL162718.1* | 84,638 | T | 0.02 | 0.573 (0.119) | 1.96×10^-6^ |
| rs11514814 | 7 | 9,104,901 | Intergenic | *AC009500.2* | 159,377 | C | 0.05 | 0.382 (0.080) | 2.25×10^-6^ |
| rs2079324 | 7 | 9,106,762 | Intergenic | *AC009500.2* | 161,238 | G | 0.05 | 0.382 (0.080) | 2.25×10^-6^ |
| rs10269163 | 7 | 9,107,209 | Intergenic | *AC009500.2* | 161,685 | A | 0.05 | 0.382 (0.081) | 2.65×10^-6^ |
| rs1014290 | 4 | 9,610,959 | Intronic | *SLC2A9* | 0 | G | 0.31 | -0.183 (0.039) | 3.03×10^-6^ |
| rs7731680 | 5 | 102,970,376 | Intergenic | *NUDT12* | -43,983 | G | 0.35 | 0.178 (0.038) | 3.61×10^-6^ |
| rs6596536 | 5 | 102,970,693 | Intergenic | *NUDT12* | -44,300 | A | 0.35 | 0.177 (0.038) | 3.71×10^-6^ |
| rs7734977 | 5 | 102,976,044 | Intergenic | *NUDT12* | -49,651 | G | 0.35 | 0.176 (0.038) | 4.48×10^-6^ |
| rs7701241 | 5 | 102,976,055 | Intergenic | *NUDT12* | -49,662 | G | 0.35 | 0.176 ( 0.038) | 4.48×10^-6^ |
| rs4529048 | 4 | 9,606,210 | Intronic | *SLC2A9* | 0 | C | 0.32 | -0.178 (0.039) | 4.63×10^-6^ |
| rs6700439 | 1 | 217,959,714 | Within noncoding | *SLC30A10* | 0 | G | 0.04 | 0.407 (0.088) | 4.66×10^-6^ |
| rs12300905 | 12 | 44,832,050 | Intergenic | *SLC38A1* | 31,060 | T | 0.12 | 0.258 (0.056) | 5.03×10^-6^ |
| rs10939650 | 4 | 9,607,538 | Synonymous | *SLC2A9* | 0 | C | 0.32 | -0.178 (0.039) | 5.14×10^-6^ |
| rs12309226 | 12 | 44,838,265 | Intergenic | *SLC38A1* | 24,845 | G | 0.12 | 0.253 (0.056) | 6.90×10^-6^ |
| rs3822242 | 7 | 9,704,002 | Intergenic | *AC009500.2* | 0 | C | 0.35 | -0.173 (0.038) | 7.33×10^-6^ |
